# Supplementary material for: Quercetin Inhibits the Proliferation and Aflatoxins Biosynthesis of Aspergillus flavus
Source: Toxins (Basel). 2019 Mar 9;11(3):154. doi: 10.3390/toxins11030154 (PMC6468572; doi:10.3390/toxins11030154)
Supplement: Supplementary file 1 [file toxins-11-00154-s001.zip › toxins-454741-supplementary.pdf]

Supplementary Materials: Quercetin Inhibits the Proliferation and Aflatoxins Biosynthesis of *Aspergillus flavus*

Xiu-Mei Li, Zhong-Yuan Li, Ya-Dong Wang, Jin-Quan Wang and Pei-Long Yang

Table S1. Summary of Transcriptome Sequencing Data.

Analysis items	Count (Million)		Proportion (%)	
	CK	QT	CK	QT
**No. of clean reads**				
Total clean reads	50561156	51441686	\	\
Q30	\	\	91.46	91.69
GC content	\	\	52.33	52.39
**Mapping to genome**				
Mapped reads	45577031	46843066	90.14%	89.88%
Uniquely mapped reads	45342143	45999155	89.68%	89.42%
Multi-mapped reads	234888	236632	0.46%	0.46%

Table S3. GO Ontology (GO) functional enrichment analysis of different expressed genes when *A.flavus* was treated with quercetin.

GO ID	Gene Ontology Term	*q* Value	List Hits	List Size
GO:0003735	structural constituent of ribosome	1.53e-48	63	339
GO:0005198	structural molecule activity	4.24e-42	63	339
GO:0009055	electron carrier activity	0.02828	8	339
GO:0019843	rRNA binding	0.03596	4	339
GO:0003755	cis-trans isomerase activity	0.04628	6	339
GO:0006412	translation	9.72e-39	75	347
GO:0044267	cellular protein metabolic process	7.27e-21	91	347
GO:0019538	protein metabolic process	5.62e-16	96	347
GO:0044249	cellular biosynthetic process	5.68e-12	116	347
GO:0009058	biosynthetic process	5.80e-12	124	347
GO:1901576	organic substance biosynthetic process	1.26e-11	117	347
GO:0044237	cellular metabolic process	3.37e-09	181	347
GO:0034645	cellular macromolecule biosynthetic process	2.94e-08	81	347
GO:0010467	gene expression	7.77e-08	83	347
GO:0009059	macromolecule biosynthetic process	7.81e-08	81	347
GO:0071704	organic substance metabolic process	6.01e-05	186	347
GO:0044238	primary metabolic process	0.00013	175	347
GO:0044260	cellular macromolecule metabolic process	0.00061	107	347
GO:0009987	cellular process	0.00863	211	347
GO:1901566	organonitrogen compound biosynthetic process	0.00952	30	347
GO:0009145	purine nucleoside triphosphate biosynthetic process	0.02823	6	347
GO:0009152	purine ribonucleoside triphosphate biosynthetic process	0.02823	6	347
GO:0005840	ribosome	9.72e-39	66	172
GO:0030529	ribonucleoprotein complex	7.27e-21	75	172
GO:0043228	non-membrane-bounded organelle	1.62e-31	73	172
GO:0043232	intracellular non-membrane-bounded organelle	1.62e-31	73	172
GO:0044444	cytoplasmic part	3.98e-29	86	172
GO:0032991	macromolecular complex	2.91e-25	93	172
GO:0005737	cytoplasm	4.54e-24	97	172
GO:0005623	cell	1.37e-09	133	172
GO:0044464	cell part	1.37e-09	133	172

GO ID	Gene Ontology Term	*q* Value	List Hits	List Size
GO:0044424	intracellular part	2.74e-08	124	172
GO:0005622	intracellular	9.83e-08	126	172
GO:0043226	organelle	2.17e-07	105	172
GO:0043229	intracellular organelle	2.17e-07	105	172
GO:0044391	ribosomal subunit	4.90e-06	11	172
GO:0015935	small ribosomal subunit	0.00051	7	172
GO:0016469	proton-transporting ATP synthase complex	0.01866	5	172

Table S4. KEGG metabolic pathway enrichment analysis of differentially expressed genes when *A. flavus* was treated with quercetin.

ID	Molecular Function	*p* Value	*q* Value	List Hits	List Size
Afv03010	Ribosome	5.971495e-55	1.009183e-52	70	318
Afv00190	Oxidative phosphorylation	2.225421e-06	1.880481e-04	20	318
Afv05016	Huntington's disease	7.707923e-06	4.342130e-04	18	318
Afv05012	Parkinson's disease	1.362634e-05	5.757129e-04	15	318
Afv05010	Alzheimmer's disease	4.953394e-05	1.674247e-03	15	318

Table S5. Primers used for RT-PCR.

Target Gene	Primer Name	Sequence (5'→3')
aflS	F	CGTCTATCAACAGCAACACAACCT
	R	GTTCCGCAACACCTCCACAT
β-tublin	F	TTGAGCCCTACAACGCCACT
	R	TGGTTCAGGTCACCGTAAGAGG
